# Case of Pott’s Disease in a 17-Year-Old Patient in the Dominican Republic

**DOI:** 10.7759/cureus.4922

**Published:** 2019-06-17

**Authors:** Janetly Reinoso, Jonathan Arias, Genara Santana

**Affiliations:** 1 Medical Department, Pontifical Catholic Univeristy Mother and Teacher, Santiago, DOM; 2 College of Medicine, University of Florida, Gainesville, USA; 3 Infectious Disease, University Hospital of Arturo Grullón, Santiago, DOM

**Keywords:** tuberculosis, pott’s disease, infection, spinal cord

## Abstract

Pott’s disease, or spinal tuberculosis, is caused by the pathogen Mycobacterium tuberculosis. Although tuberculosis is endemic in developing countries, such as Dominican Republic, Pott’s disease is rarely seen, only representing 1% of the total cases. Due to its low incidence, this could be easily misdiagnosed. We report a case of a 17-year-old male with a history of pleural effusion that presented with severe back pain. Several imaging studies reported an aggressive paravertebral neoplasia at the thoracic levels. A surgical biopsy was performed, and the procedure revealed bone fragmentation, which prompted the need to rule out Pott’s disease per current recommendations. Biopsy and subsequent positive QuantiFERON-TB Gold test confirmed spinal tuberculosis.

## Introduction

Spinal tuberculosis (ST), or Pott’s disease, was first described by Sir Percival Potts in 1779, albeit lacking a deep understanding of its etiology or pathophysiology. It was only after several years that the basis of ST was discovered to be the bacteria currently known as Mycobacterium tuberculosis [1]. Tuberculosis (TB) is considered one of the worldwide leading causes of death, making it a global health burden [2]. Developing countries, such as Dominican Republic (DR), have an increased incidence of TB. For example, it is estimated that 60 cases per 100,000 are diagnosed each year in the DR [3]. However, ST represents less than 1% of the total of cases of TB and accounts for 10% of extrapulmonary tuberculosis [4,5]. Therefore, it is recommended to medical providers to rule out TB when encountering a spinal lesion. Here, we report a case of Pott’s disease in the Dominican Republic.

## Case presentation

**Figure 1:**
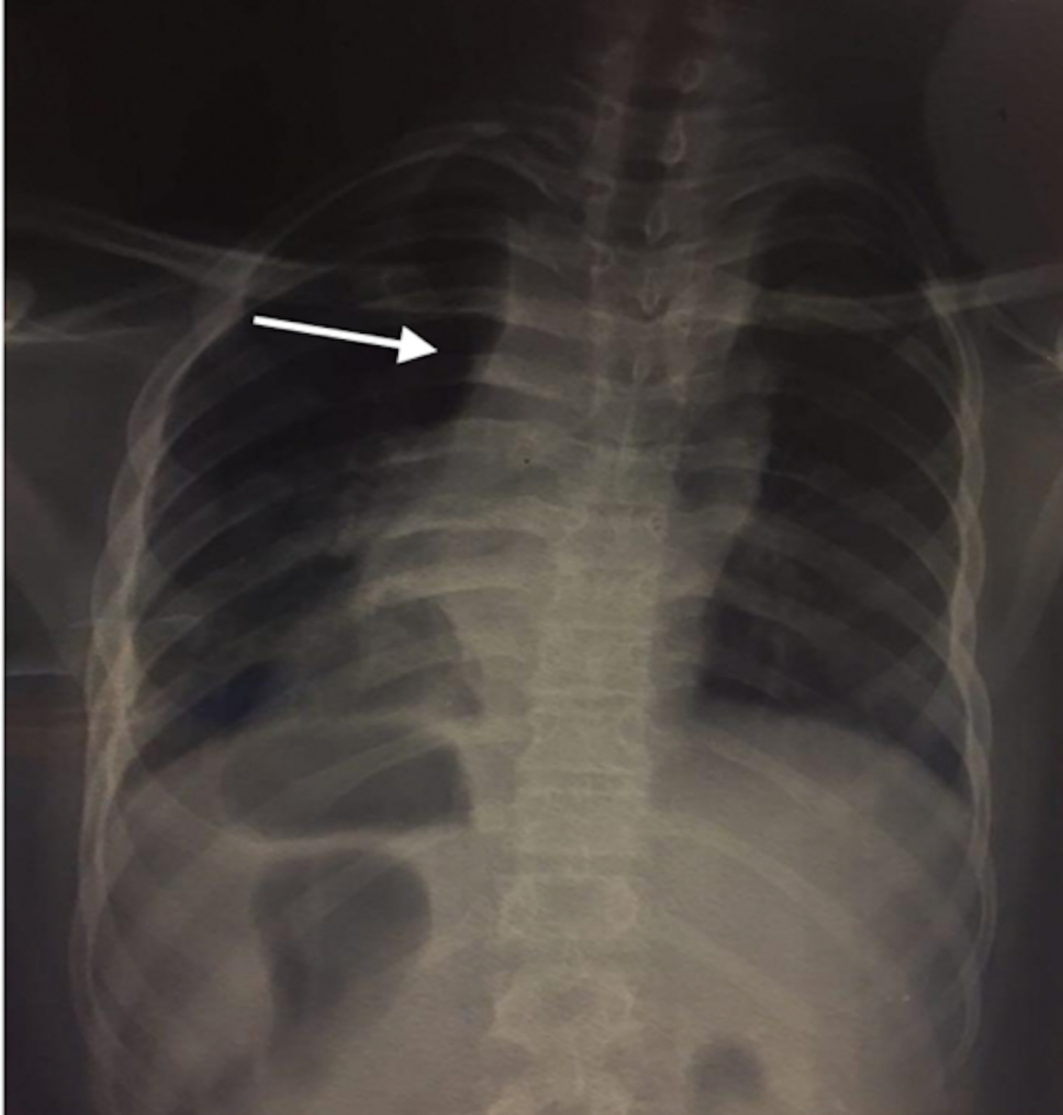
Chest X-ray Showing mediastinal widening.

**Figure 2:**
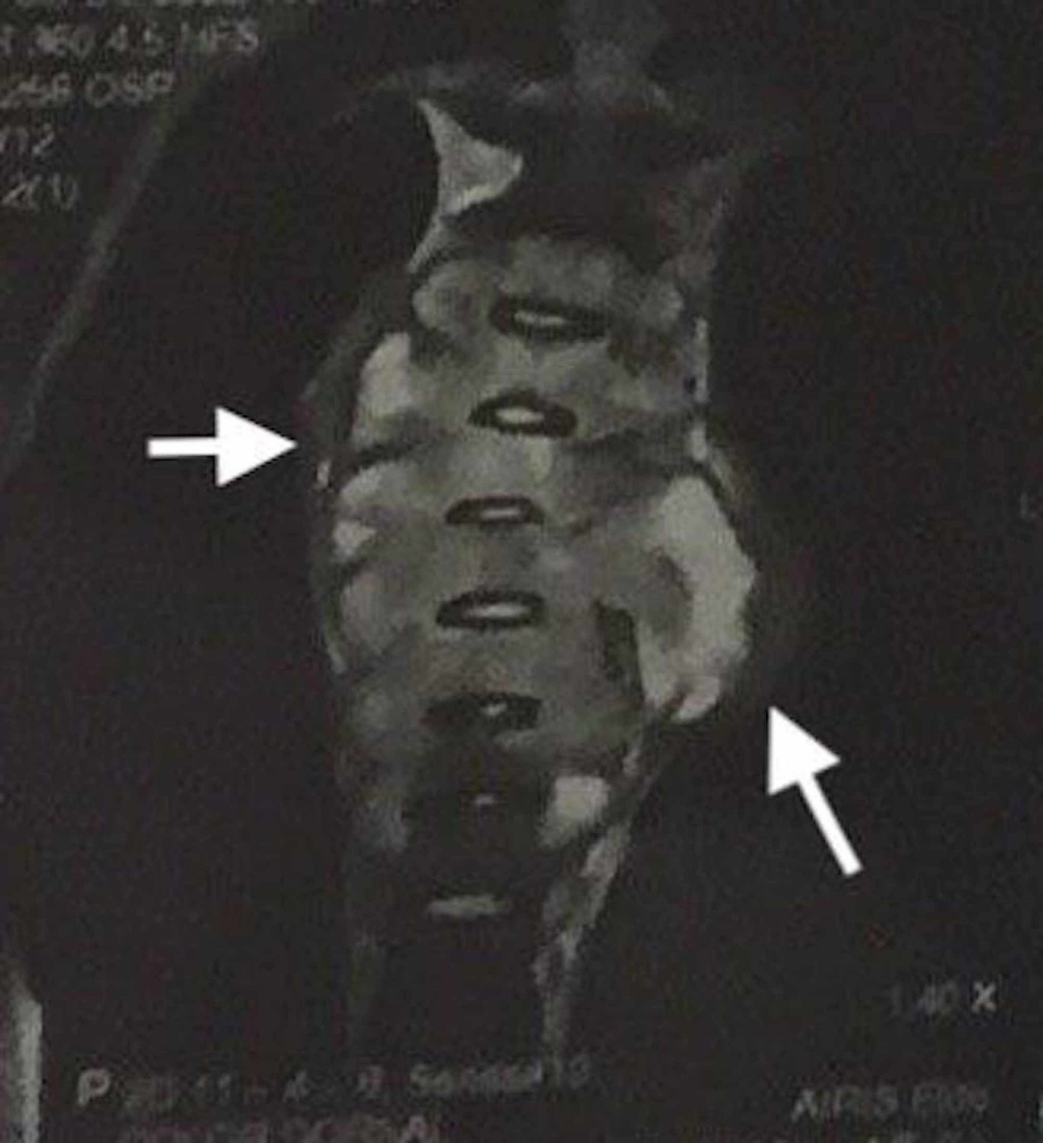
MRI coronal view Showing large paravertebral lesion with cystic appearance and an infiltration of the vertebral bodies.

**Figure 3:**
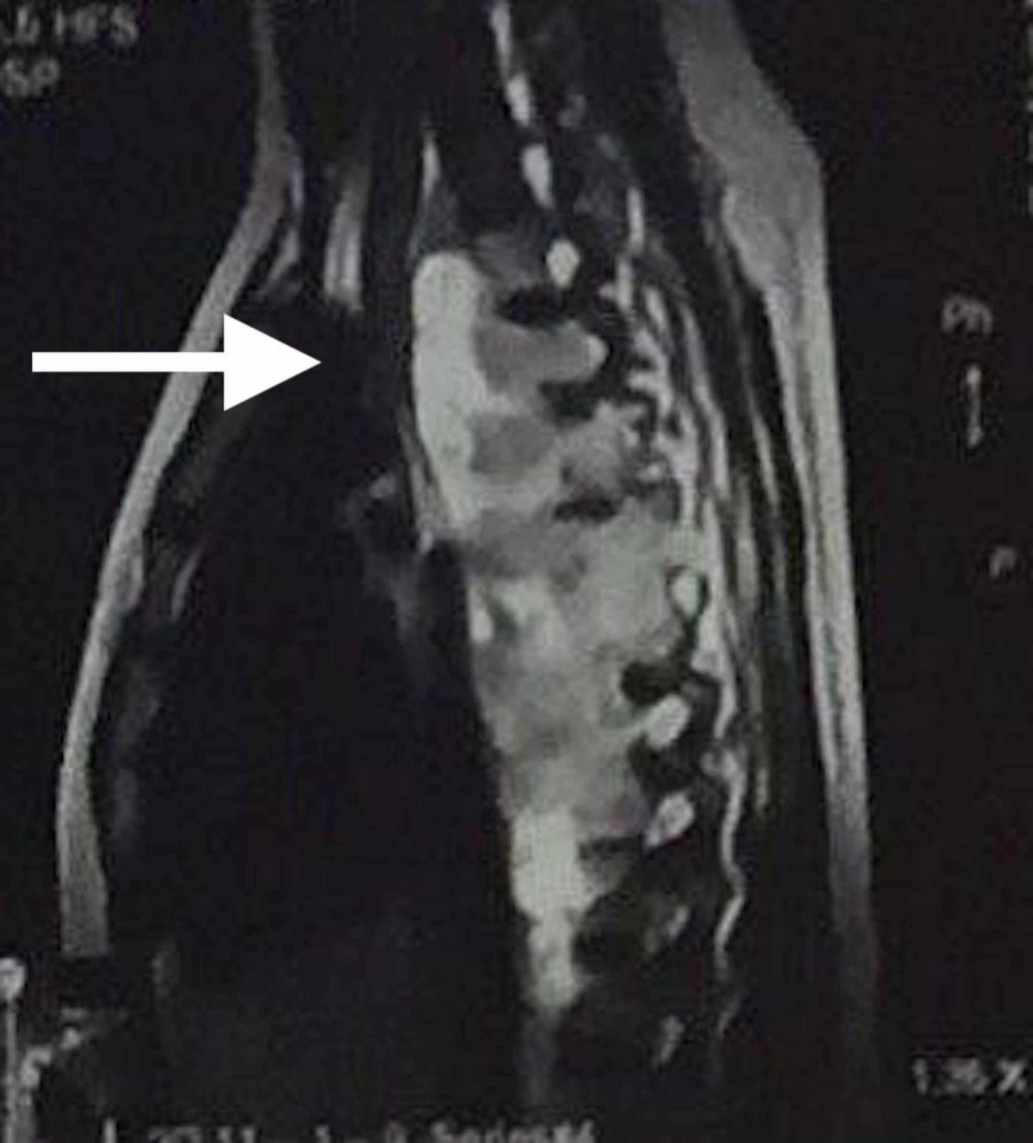
MRI sagittal view Showing paravertebral lesion, infiltration of the vertebral bodies.

A 17-year-old male presented to the emergency department of Hospital Universitario Arturo Grullón in the Dominican Republic complaining of localized thoracic back pain for the past week. The patient described the pain as progressively worsening, increasing from a rating of 3/10 to a rating of 10/10. He had a history of left-sided pleural effusion that was managed by another unrelated hospital via thoracocentesis a month prior. There was no history of trauma that could have otherwise explained the pain. The focused neurological physical examination was completely benign, except for pain upon passive and active range of motion of his back; deep tendon reflexes at the patellar and achilles tendons were +2, gait was steady, straight leg test was negative, and sensation to the lower extremities upon light touch and pinprick was normal and symmetrical. Due to the severity of the pain and prior history of pleural effusion, a chest X-ray and CT scan were performed. The chest X-ray showed mediastinal widening (Figure 1), and the CT scan reported a paraspinal neoplasia in the posterior mediastinum from T1-T10 with regular margins and little vascularity that did not infiltrate the dorsal column nerve structure. Upon MRI, a large paravertebral lesion with cystic appearance and infiltration of the vertebral bodies was seen, indicating an aggressive neoplasia (Figures 2, 3). Of noteworthy is that tumor markers were ordered with hopes to support a diagnosis of presumptive neoplasia, but was negative; and during a following surgical biopsy bone fragments were observed and palpated in the vertebral bodies, suggestive of osteomyelitis and prompting Pott’s disease in the differential. Anti-tuberculosis treatment consisting of two months of Isoniazid, Rifampin, Ethambutol, and Pyrazinamide, followed by four months of Isoniazid and Rifampin was initiated prior to laboratory and biopsy results. The patient was noticed to have significant clinical improvement of symptoms within four weeks. A smear microscopy and Xpert MTB-RIF tests were ordered and were negative; and a QuantiFERON-TB Gold test was also ordered, which resulted positive. Likewise, the biopsy reported a chronic inflammatory granulomatous process with extensive areas of necrosis, suggestive of tuberculosis. Despite the findings, the patient was always stable and without gait disturbance. The previously mentioned anti-tuberculosis therapy was continued, with significant improvement of symptomatology. He was discharged on the 9th day of his admission. The patient was referred to the national tuberculosis control program for continuation of treatment.

## Discussion

The Dominican Republic is one out of six countries with the highest rates of tuberculosis in Latin America [6]. Nonetheless, spinal tuberculosis only represents 1% of all cases [3,4]. This disease is rarely seen and misdiagnosis can occur [1]. The primary infection site typically comes from either a pulmonary focus or other extrapulmonary foci that may be active or latent [7]. Our patient had a history of pleural effusion for which he was admitted a month prior, and was managed by a thoracocentesis at another hospital; however, there was no indication that screening for TB was performed. Unfortunately, due to the logistical challenges in communication and sharing of medical records amongst different hospitals in a medical system without electronic medical records, the results and other tests associated with our patient's pleural effusion are unknown. In any case, we believe that the etiology of our patient’s spinal tuberculosis was an extrapulmonary focus in the form of a pleural effusion.

The clinical presentation of ST is highly variable and depends on how advanced the disease is. Usually, its presentation is very nonspecific and insidious. The most common symptoms are back pain, fever, weight loss, night sweats, and fatigue [8]. In the present patient, he only presented with back pain, which is observed to be the only symptom in 61% of cases of spinal tuberculosis [9]. The absence of constitutional symptoms, such as fever and fatigue, causes clinicians to mistakenly exclude TB as a possible cause of back pain [10]. This can potentially delay diagnosis and treatment, which may result in devastating neurological complications such as syringomyelia and paraplegia [11]. Definitive diagnosis of ST requires the presence of both clinical suspicion and imaging findings. Although MRI is considered a valuable diagnostic tool for ST, it cannot differentiate between infection or malignancy [12]. As seen with our patient, both MRI and CT imaging could have been misleading as it demonstrated an invasive neoplasia. If not for the observed bony fragments suggestive of osteomyelitis made during the surgical biopsy, suspicion for ST would not have been considered and specific test for it would have been further delayed or possibly overlooked. This highlights the importance of maintaining a broad differential and performing non-overlapping studies to cover as many etiologies possible in order to avoid erroneous diagnosis and bad outcomes.

There are several recommended tests indicated for spinal tuberculosis, or Pott’s disease. According to Rajasekaran et al., Xpert MTB-RIF is the most useful, especially when a smear microscopy is negative [13]. In this case both the smear and the Xpert MTB-RIF were negative. However, the QuantiFERON-TB Gold was positive and as previously mentioned subsequent biopsy confirmed the diagnosis. As suggested by current guidelines, appropriate treatment allocation was started immediately before any tests or culture results were available due to the high clinical suspicion following the observations made during the surgical biopsy [1].

Treatment of ST can be conservative, surgical, or both. Its management can be classified as either with or without neurological deficit. Patients without neurological should be treated with a more conservative approach (i.e., medical therapy), with few cases requiring surgery. Patients with neurological deficits have better outcomes when managed with a combination of both medical therapy and surgical intervention [14]. Our patient had no neurological compromise, thus, he was solely treated conservatively with medical therapy. According to the national guideline for tuberculosis, patients with extrapulmonary tuberculosis should initiate 2HRZE/4HR and be followed by the national tuberculosis control program for continuation of treatment [6]. Upon initiation of such treatment, the patient showed significant improvement and was relieved of his symptoms.

## Conclusions

Spinal tuberculosis can mimic other diseases, such as a neoplasia. Because back pain is the most common and sometimes only presenting symptom, misdiagnosis is fairly common. It is paramount to maintain a broad differential diagnosis as clinicians, and is especially important in rare disease such as spinal tuberculosis (ST). This facilitates earlier diagnosis and treatment, leading to improved health outcomes and avoidance of any potential complications.
